# Researches on the Tubercular Disease of the Bones

**Published:** 1839-07-27

**Authors:** 


### BIBLIOGRAPHICAL NOTICE.

*Researches on the Tubercular Disease of the Bones.* By A. Nelaton, M. D. Translated from the French, by Charles R. King, M. D.

The Children's Hospital of Paris is the grand receptacle for the scrofulous children of the poorer inhabitants of Paris. The porters, the inhabitants of the dark and damp dwellings of the citè, and of the Faubourgs St. Victor and St. Marceau, are cooped up in unwholesome dwellings, with little light and air, and food of the poorest quality; a necessary consequence is, that numbers of their children are attacked with scrofulous caries of the bones. During our residence at Paris we observed many dissections of these diseased bones at the Children's Hospital, made by Dr. Rufz, now of Martinique; these dissections showed that scrofulous caries of the bones might be considered nearly, if not quite identical with tuberculous diseases. Softening of the tuberculous matter takes place in the usual way, and caries results.

Dr. Nelaton succeeded Dr. Rufz as interne in the surgical service of the hospital, and prosecuted still further the examination of the subject. The results which he obtained were published, and we are indebted to Dr. King, of New York, for the translation of Dr. Nelaton's memoir.

We extract the more interesting parts of Dr. Nelaton's memoir. One remark we may make, is, that all these diseases are much more fatal at the Children's Hospital than at other institutions, owing to the peculiar constitution of the patients, and, perhaps, the vitiation of the air, caused by the large number of children there collected, notwithstanding the scrupulous cleanliness of the institution.

The following table, in which I have brought together the principal traits of each of these two forms of tubercular disease in their order of succession, will serve as a summary of what has been previously said, and place in bold relief the differences between them.

ENCYSTED TUBERCLES.

1st. Semi-transparent gray granulations.
2d. Crude, opaque and encysted tubercle.
3d. Bony excavation, loss of substance in that tissue.
4th. Evacuation of the tubercular cavity.
6th. Hypertrophy of the cyst, obliteration of the cavity, recovery.

In this table I have represented only the most simple march of the disease, and the termination towards which it appears naturally to tend. But it has already been seen, and will be still more evident in the second part of this work, how local and accidental circumstances may interfere with this favourable result.

TUBERCULAR INFILTRATION.

1st. Semi-transparent gray infiltration.
2d. Interstitial hypertrophy of the bony tissue.
3d. Puriform infiltration.
4th. Necrosis of the infiltrated portion.
5th. Sequestration. Foreign body.

It may cause a little surprise to find in the preceding table the interstitial hypertrophy of the bony tissue placed between the semi-transparent gray infiltration and the puriform; my intention is by it to indicate that I consider the hypertrophy as an intermediate phenomenon between these two states, a phenomenon which manifests itself during the passage from the first to the second degree; for we have seen that this hypertrophy does not exist during the first period, while it cannot be admitted to take place during the second, when the bony tissue is deprived of its elements of nutrition and of life.

From what has been said, it is clear that the first variety of the tubercular disease could not be confounded with any other affection of the bony tissues; that it has its peculiar march and physiognomy, and affords but very distant analogies with the other organic lesions of this tissue. As to the second variety, it is not at all astonishing that it has been so long, and is still daily, confounded with caries; a mistake arising from the want of positive precision as to the anatomical character of caries, and from the fact that under this name very different pathological conditions are described. Besides, it must be allowed, that a portion of cellular tissue infiltrated with softened tubercular matter has, indeed, some resemblance in aspect to a carious portion; but it will always be easy to recognise the puriform tubercular infiltration by the interstitial hypertrophy, by the augmentation of solidity, and by the absence of vascularity; whilst in caries, properly so called, there is a rarefaction, a softening, and an increased vascularity of the bony tissue. Consequently, they are distinguished by organic conditions, not only differing from, but directly opposed to, each other. Caries always proceeds from the circumference to the centre of the bone; the tubercular disease from the centre to the circumference. Nothing would be more easy than to accumulate points of distinction between these two diseases; but I think enough has been said to avoid confounding them.

It is much more easy to understand its being mistaken for a simple necrosis; but, in a given case, the nature of the organic lesion will be easily determined, by recollecting that simple necrosis does not cause any modification of the intimate structure of the dead tissue, whilst the tubercular infiltration causes before-hand an interstitial hypertrophy.

There is still another pathological condition of the bones with which tubercular infiltration is confounded; I refer to the suppuration of that tissue, a disease described under the name of inflammation of the medullary membrane of the bones; the interstitial hypertrophy will suffice here, also, to prevent the mistake.

### SEAT OF THE TUBERCULAR DISEASE.

Although I have not treated separately of the seat of the two varieties of this disease, it will be easy to recognise, from the data we have passed in review, what is common to both, and what is peculiar to one or the other. All parts of the bony system are not equally liable to its attack; it may indeed be said, in a general manner, that it developes itself almost always in the spongy tissue of the bones. But we must remark here, that the spongy tissues of the bones, in an adult, presents itself under two different conditions, which may be designated by the names of *adipose* cellular tissue, and of *vascular*, *bloody*, or simply *red* cellular tissue. The first variety constitutes the extremities of the long bones and the short bones of the limbs. All the bones of the trunk are formed of the second; to ascertain this difference, it is only necessary to compare the spongy tissue of one of the condyles of the femur with that of the body of a vertebra in the adult. In the first, the meshes are filled with a yellow, adipose matter; while the cells of the body of the vertebræ are composed of an extremely vascular red substance, presenting scarcely any trace of fatty matter; now, it is in this second variety of the cellular tissues of the bones that the tubercular productions are almost exclusively developed. In children, as this difference in the tissues does not exist, and as the bones of the limbs, as well as those of the trunk, are all formed of this red cellular tissue, the tubercular disease attacks indifferently the bones of the limbs and of the trunk; in the adult, on the contrary, it is met with only in the bones of the trunk. This law admits of but very few exceptions; these too could perhaps be explained by the fact of a tardy transformation of the red cellular tissue into the adipose. It is in the very centre of the bony tissue, rather than at its surface, that this disease originates; a fact more easily ascertained in the case of encysted tubercles than in that of tubercular infiltration, which being ordinarily diffused, speedily reaches the surface of the bone. In the long bones, the extremities are the most usual seat of tubercles; and it has appeared to me, that they are more frequently developed in the epiphysis than in the contiguous and enlarged extremity of the diaphysis. Still, I have met with some cases, rare, it is true, of encysted tubercles lodged in the compact tissue of the diaphysis; but I could not affirm that the accidental production was not developed in the medullary tissue, and that the destruction of the compact tissue was not consecutive. In the long bones, one extremity is generally attacked in preference to the other; thus in the femur, it is the inferior extremity; the contrary is the case with the tibia; in the humerus and the bones of the fore-arm, the cubital extremity is most generally affected.

This organic lesion has been observed in almost every bone of the skeleton; the following classification will indicate the order of frequency in each of them:

1st. Vertebræ;
2d. Tibia, femur, humerus, (in children;)
3d. Phalanges, metatarsal metacarpal bones;
4th. Sternum and ribs, iliac bones;
5th. Petrous portion of the temporal bone;
6th. Short bones of the tarsus and of the carpus.

The bones not mentioned in this table should be classed with those with which they have the greatest analogy of form and structure.

From the preceding data, we may conclude that the two varieties of the tubercular disease of the vertrebræ differ in a marked manner from each other, in respect to their essential characteristics, and in respect to their severity. The first alone seems to me susceptible of cure; the second is always incurable, because it generally attacks a greater extent of the vertebral column, and especially, on account of the sequestra, veritable foreign bodies, which keep up a deep and inexhaustible suppuration. It will now be seen at once, how we explain that opinion, which at first appeared a true surgical paradox, namely: that the profound caries is alone susceptible of cure, while superficial caries is ordinarily incurable. The first announces itself by a gibbosity, which appears sometimes suddenly, but always very rapidly; the second by a deformity that manifests itself only after some time, and in an insensible manner. Thus, all other things being equal, the sudden appearance of a gibbosity, in a case of vertebral disease, should be considered rather a fortunate than an unfavourable circumstance; for it is, in our estimation, the indication of the existence of the first form of this affection, which alone, as we have said, is susceptible of cure.

*Tubercles in the articular extremities of the long bones.*—The tubercular disease seated in the spongy extremities of the long bones, and consequently in the vicinity of the articulations, gives rise to various pathological phenomena, generally comprehended under the denomination of white-swelling. These phenomena should be separately studied in each of the two varieties of the tubercular disease of the bones. I will not here speak of the secondary symptoms of these white-swellings, such as pain, engorgement of the tissues surrounding the articulation, tubercular abscesses, and the fistula succeeding them; they would belong to a complete history of the tubercular white-swelling; I shall dwell solely upon two points which may be considered as characterizing these two varieties.

When an encysted tubercle is developed in the extremity of a long bone, it is at first enclosed in the middle of the spongy tissue of the bone, at a small distance from the articular cavity; it increases in all directions, and gradually approaches, on the one side, the articular cavity, and on the other, the periphery of the bone situated outside of the articulation. If, in the progress of development, it reaches more quickly the surface outside of the articulation, it empties itself into the neighbouring cellular tissue; an abcess forms there, grows and opens, and is followed by a fistula; the tubercular cyst becomes hypertrophied, the cavity in the bone is filled up, and a spontaneous cure is common in this case. But if, on the contrary, the tubercle first reaches the articulation, either because it has commenced at but a little distance from it, or because the new bony layers, accumulating where the bone is covered by its periosteum, cause the constant retrogradation, so to speak, of the periphery of the bone, the diarthrodial cartilage is perforated, the tubercular matter is effused into the articulation, and suddenly occasions an intense arthritis, which is accompanied with the most serious local and general symptoms, and may be followed by an almost certain disorganization of all parts of the articulation."—*N. Y. Journ. of Med. & Surg.*

